# Making routine native SAD a reality: lessons from beamline X06DA at the Swiss Light Source

**DOI:** 10.1107/S2059798319003103

**Published:** 2019-03-12

**Authors:** Shibom Basu, Aaron Finke, Laura Vera, Meitian Wang, Vincent Olieric

**Affiliations:** a Swiss Light Source, Paul Scherrer Institut, Villigen PSI, Switzerland; b MacCHESS, Cornell University, Ithaca, New York, USA

**Keywords:** native SAD phasing, anomalous signal, data-collection strategy, multi-axis goniometer, data multiplicity

## Abstract

Native single-wavelength anomalous dispersion (SAD) is the most attractive *de novo* phasing method in macromolecular crystallography, as it directly utilizes intrinsic anomalous scattering from native crystals. However, the success of such an experiment depends on accurate measurements of the reflection intensities and therefore on careful data-collection protocols. Here, the low-dose, multiple-orientation data-collection protocol for native SAD phasing developed at beamline X06DA (PXIII) at the Swiss Light Source is reviewed, and its usage over the last four years on conventional crystals (>50 µm) is reported. Being experimentally very simple and fast, this method has gained popularity and has delivered 45 *de novo* structures to date (13 of which have been published). Native SAD is currently the primary choice for experimental phasing among X06DA users. The method can address challenging cases: here, native SAD phasing performed on a strept­avidin–biotin crystal with *P*2_1_ symmetry and a low Bijvoet ratio of 0.6% is highlighted. The use of intrinsic anomalous signals as sequence markers for model building and the assignment of ions is also briefly described.

## Abstract 2

A broadly applicable, simple and fast data-collection strategy for native SAD is described, which has become the primary choice for experimental phasing among users of the macromolecular crystallography beamline X06DA (PXIII) at the Swiss Light Source. Its usage over the last four years is reviewed.

## 1. Introduction   

Single-wavelength anomalous dispersion (SAD) is the most popular experimental phasing technique for *de novo* structure determination by macromolecular crystallography (MX). Nearly 50% of the novel structures deposited in the Protein Data Bank (PDB; Berman *et al.*, 2000 ▸) were determined using this method. The vast majority of these SAD experiments exploited the strong anomalous signal of heavy atoms, delivered either by soaking of heavy elements or by the expression of selenomethionyl protein (Hendrickson, 2014 ▸). However, these derivatizations can be problematic owing to loss of both isomorphism and diffraction. Thus, a SAD experiment using only native crystals is very appealing. This method, called native SAD, which exploits the weak anomalous signals from light elements (*Z* < 20, *i.e.* mostly S, P, Cl^−^, K^+^ and Ca^2+^) that are natively present in biomolecules, has certain challenges that have, until recently, kept it from being a general method. The anomalous signal of these light scatterers is higher at low energies (<6 keV), but X-ray absorption from the sample, including the crystal, the surrounding solvent and the mounting medium, and especially the absorption of X-rays by air lead to a significant attenuation of the diffracted signals. Other difficulties in low-energy crystallography are on the detector side, with both inaccurate calibration and a loss of high-angle diffraction with a typical flat configuration, owing to the increasing Bragg angles of reflection. To overcome these limitations and push the limit of native SAD phasing (Bent *et al.*, 2016 ▸), special setups designed for native SAD data collection at energies below 5 keV have been developed at beamlines I23 at Diamond Light Source in the UK (Wagner *et al.*, 2016 ▸) and BL-1A at the KEK Photon Factory in Japan (Liebschner *et al.*, 2016 ▸). Provided that diffraction extends to ∼2.8 Å resolution, high-quality data for native SAD phasing can also be obtained on conventional macromolecular crystallography beamlines using an energy of 6 keV (Mueller-Dieckmann *et al.*, 2007 ▸; Liu *et al.*, 2012 ▸; Weinert *et al.*, 2015 ▸).

The evolution of native SAD as a method has recently been reviewed (Rose *et al.*, 2015 ▸; Rose & Wang, 2016 ▸; Liu & Hendrickson, 2017 ▸). The first example of the *de novo* SAD phasing of a native protein was performed in 1981 by Hendrickson and Teeter using crambin (46 residues with six S atoms; Hendrickson & Teeter, 1981 ▸). However, this was an especially remarkable case owing to the high resolution and stability of crambin crystals and, while no other structure was solved by native SAD for over a decade afterwards, this report opened the doors to phasing by heavy-atom SAD. Essentials in making SAD a practical experiment were then advances in substructure determination, solvent flattening and density modification (Wang, 1985 ▸). Early experimental protocols for anomalous phasing called for carefully designed measurements from an aligned crystal along an evenfold symmetry axis, which was useful in measuring Bijvoet pairs with minimum dose difference (Hendrickson, 2014 ▸). Similarly, wedges of data 180° apart were collected in the so-called ‘inverse-beam’ method (Hendrickson *et al.*, 1985 ▸). The benefit of high multiplicity was subsequently demonstrated for native SAD phasing (Dauter *et al.*, 1999 ▸; Dauter, 1999 ▸; Dauter & Adamiak, 2001 ▸; Debreczeni, Bunkóczi, Girmann *et al.*, 2003 ▸; Debreczeni, Bunkóczi, Ma *et al.*, 2003 ▸; Debreczeni, Girmann *et al.*, 2003 ▸; Mueller-Dieckmann *et al.*, 2004 ▸); nonetheless, a simpler and faster protocol was common practice at high-throughput third-generation synchrotron MX beamlines. Optimized for CCD-based detector technology, it consisted of collecting the minimum rotation range required to achieve 100% completeness, usually with a high X-ray dose. Performed using a single-axis goniometer, this protocol was also practiced for experimental phasing, and while it may suffice for experimental phasing with strong scatterers such as selenium, the deleterious effects of radiation damage make it inadequate for native SAD except for cases of high resolution and high symmetry. Meanwhile, multi-axis goniometers, which are standard in the world of small-molecule crystallography, and also used to be the norm for MX 30 years ago, have largely been replaced on MX beamlines by single-axis, air-bearing goniometers, which feature submicrometre spheres of confusion and rapid rotation rates, and they have done so with great success. However, as beamline hardware evolved without the demand for multi-axis goniometry, it became clear that older goniometer designs were not compatible with new beamlines, and were certainly not precise enough to handle the ever-decreasing size of protein crystals. However, the loss of multi-axis goniometry also meant losing the ability to freely reorient samples in a controlled manner. Why does this matter? There are two main advantages to multi-orientation data collection that turn out to be critical for native SAD. Firstly, collecting data in multiple orientations means that reflection intensities are measured multiple times on different areas of the detector, which averages out systematic errors. Secondly, nonspherical crystals experience orientation-dependent absorption, which can be more effectively modelled by collecting data in multiple orientations. Thus, multi-orientation data collection leads to more accurate intensity measurements, which mean more accurate anomalous difference estimations. In cases where the measured anomalous difference is 1% or less of the total intensity, as is typical for native SAD, high accuracy is obviously very important.

In 2011, Liu and Hendrickson showed the power of multi-crystal averaging to boost the anomalous signal (Liu *et al.*, 2011 ▸) and applied it to native SAD by carefully selecting and merging statistically equivalent data sets over multiple crystals collected at a tolerable dose of ∼5 MGy (Liu *et al.*, 2012 ▸, 2013 ▸, 2014 ▸; Liu & Hendrickson, 2015 ▸). More challenging examples followed (El Omari *et al.*, 2014 ▸; Akey *et al.*, 2014 ▸, 2016 ▸; Zander *et al.*, 2015 ▸). A similar approach consisting of collecting multiple low-dose data sets (<0.5 MGy per 360°) in multiple orientations proved to be successful in the challenging case of native SAD using only one crystal (Weinert *et al.*, 2015 ▸). The latter strategy, which is reviewed in detail in this article, was realized using the multi-axis goniometer PRIGo (Parallel Robotics Inspired Goniometer; Waltersperger *et al.*, 2015 ▸), a new type of goniometer which combines the high precision of air-bearing goniometers with a higher flexibility for spatial reorientation.

Arguably the greatest advance in the field of MX data collection in the past decade has been the introduction of hybrid (pixel-array) photon-counting detectors (HPCs; Broennimann *et al.*, 2006 ▸), such as PILATUS (Henrich *et al.*, 2009 ▸), which are fast and have zero readout noise, and which have revolutionized MX data-collection protocols and allowed users to exploit the speed and high flux of third-generation synchrotrons. Extremely short dead and readout times, coupled with single-photon-counting sensitivity, have enabled ‘shutterless’ data collection, drastically shortening the data-collection time compared with previous CCD detectors (Mueller *et al.*, 2012 ▸). A high-quality single data set of 360° total angular range can now typically be collected in less than a minute: a huge improvement over the ∼30 min or more that this would take on a CCD detector. Aside from the fact that users could now collect hundreds of data sets in the course of a single synchrotron beam allocation (typically 8 h), it also meant that more ‘advanced’ data-collection strategies, such as multi-crystal or multi-orientation strategies, could easily be implemented and practiced routinely on MX beamlines. Further advancements in detector technology and the arrival of even faster detectors such as EIGER and JUNGFRAU (Casanas *et al.*, 2016 ▸; Leonarski *et al.*, 2018 ▸) have pushed the limit of data-collection speed, and have enabled more accurate anomalous and serial crystallographic measurements at synchrotrons (Diederichs & Wang, 2017 ▸).

Here, we review the optimized native SAD data-collection strategy (Weinert *et al.*, 2015 ▸; Olieric *et al.*, 2016 ▸) developed at the X06DA (PXIII) beamline at the Swiss Light Source (SLS) and our success with this technique since the installation of the multi-axis goniometer PRIGo (Waltersperger *et al.*, 2015 ▸). The strategy for experimental native SAD will be described, as well as the successes, failures and challenges over the course of last four years.

## 2. Materials and methods   

### 2.1. Experimental setup at the X06DA (PXIII) beamline   

The versatile X06DA (PXIII) beamline is the dedicated experimental phasing beamline for conventional crystals (>50 µm) at the SLS. The superbend magnet X-ray source provides variable photon energy (5–17.5 keV) with very high stability and with a current average flux of ∼3 × 10^11^ photons s^−1^ at 12.4 keV and 1.5 × 10^10^ photons s^−1^ at 6 keV. The beam size is fixed to 90 × 50 µm (horizontal × vertical, full width at half maximum). At 6 keV, we aim for a dose of ∼0.5 MGy per 360° data set. A double channel-cut monochromator (DCCM) enables fast and stable energy change in a short time, usually around 30 s. The beamline is equipped with the in-house-developed multi-axis goniometer PRIGo (Waltersperger *et al.*, 2015 ▸), which features a compact design and micrometre-precision rotation around the data-collection ω axis. It emulates an Eulerian arc that is described by ω, χ and φ axes (ω, infinite; χ, 0–90°; φ, 0–360°). PRIGo is used for native SAD data collection with a χ angle of up to 40°, which provides both collision-free and reduced self-shadowing when collecting diffraction data around ω. The beamline is equipped with a PILATUS 2M-F detector, which has a readout speed of 60 Hz and is calibrated for low energies. Native SAD 6 keV measurements are performed in an air environment without changes to the experimental setup. In addition, X06DA features advanced data-collection protocols with inverse beam and crystal alignment, as well as energy interleaving (Finke *et al.*, 2016 ▸). While such advanced strategies may be useful for SAD and MAD protocols with heavy atoms, low-dose data collection in multiple random crystal orientations using a modern HPC detector was found to be highly effective for native SAD (Weinert *et al.*, 2015 ▸; Olieric *et al.*, 2016 ▸) and was readily adopted by the users thanks to its operational simplicity.

### 2.2. Data-collection strategy for native SAD phasing   

**Figure 1:**
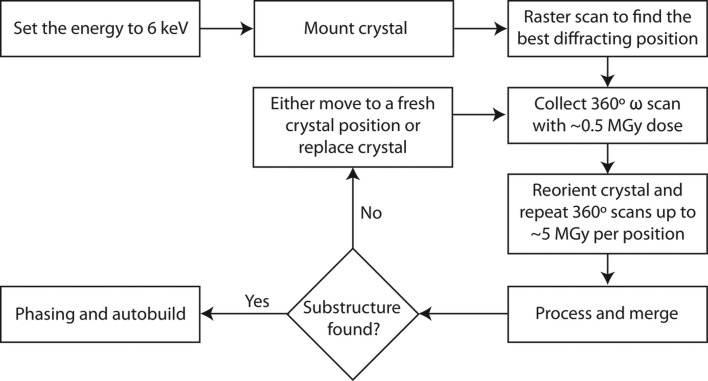
Flowchart of the low-dose multi-orientation native SAD data-collection strategy.

A workflow of the data-collection strategy for a native SAD experiment is shown in Fig. 1 ▸. It assumes that the users have screened their crystals and identified a good diffracting crystal before considering the native SAD strategy; a resolution of better than ∼2.8 Å is usually required. The choice of energy is critical for successful native SAD phasing, as mentioned in Section 1. An energy must be chosen that gives sufficient anomalous signal while minimizing the absorption from air, which becomes significant in the tender X-ray region. With the current setup at X06DA, we use an energy of 6 keV, which provides a good compromise between flux, anomalous signal and absorption (Mueller-Dieckmann *et al.*, 2005 ▸; Liu *et al.*, 2012 ▸; Weinert *et al.*, 2015 ▸). Higher energies give better resolution at the cost of decreased anomalous signal, while lower energies become increasingly affected by air and sample absorption, and thus weaker reflection intensities. A conventionally sized crystal (*i.e.* in the tens to hundreds of micrometres) is mounted onto the PRIGo multi-axis goniometer under a nitrogen cryostream set to 100 K. A raster grid scan can be used to find the best diffracting position on the crystal (Cherezov *et al.*, 2009 ▸; Bowler *et al.*, 2010 ▸; Aishima *et al.*, 2010 ▸; Hilgart *et al.*, 2011 ▸; Wojdyla *et al.*, 2016 ▸). A typical native SAD experiment involves collecting 360° ω scans at varying values of χ and φ, typically starting at χ = 0° and φ = 0°, and then collecting additional 360° ω scans in 5° χ increments. We aim for an estimated dose of ∼0.5 MGy per 360° ω scan in most cases. A warning: this typically leads to, at least at first glance, weak-looking diffraction spots when visually analyzing individual frames. This is intentional; minimizing dose and thus radiation damage is key, and scaling and merging individual low-dose data sets together gives data that are of higher accuracy and quality than a single high-dose data set (Olieric *et al.*, 2016 ▸). Provided that the crystal is homogeneous, which can be checked by rastering the crystal with a grid scan, the merging of multiple data sets from one crystal is usually straightforward. At a rate of 2° s^−1^, a 360° ω scan takes 3 min. A total of 8 × 360° ω scans are typically collected in less about 25 min from one crystal position, yielding sufficient data to obtain a good-quality solution for native SAD in all but the most challenging cases. If the data prove insufficient to generate a solution, and it is clear that there is a buildup of anomalous signal in the scaled and merged data, additional data sets from another spot on the same crystal, or from a new isomorphous crystal, can be collected and scaled together. This may be necessary in challenging cases owing to low symmetry (*i.e.* monoclinic or triclinic), insufficient resolution (∼3 Å), small crystals (∼50 µm in the longest direction) or samples that are especially prone to radiation damage.

As we alluded to above, it is desirable to process data ‘on-the-fly’ during collection in order to monitor radiation damage to the crystal and to determine when sufficient data have been collected. While this is far from an automated process, new beamline data-reduction routines do streamline it. We have developed an automated data-processing system that runs silently in online mode and provides real-time feedback via a user-friendly HTML-based web tracker (Wojdyla *et al.*, 2018 ▸). Our online software harnesses *XDS* (Kabsch, 2010 ▸) for processing individual data sets. Once a few sweeps have been processed by *XDS*, the XDS_ASCII.HKL file from each data set can be scaled and merged using *XSCALE* (Kabsch, 2010 ▸). It is useful to monitor the scaled relative *B* factors per data set given by the *XSCALE* output; high values indicate radiation damage and loss of anomalous signal, and the sample should be repositioned or replaced. If sufficient anomalous signal along with good-data quality are obtained after scaling with *XSCALE*, substructure determination can be started using *SHELXD* (Sheldrick, 2010 ▸). Once the substructure has been determined, data can be passed to phasing programs such as *SHELXE* (Sheldrick, 2010 ▸), *CRANK*2 (Skubák & Pannu, 2013 ▸), *autoSHARP* (Vonrhein *et al.*, 2007 ▸) or *AutoSol* (Terwilliger *et al.*, 2009 ▸), which combine density modification and automatic model building. If substructure determination fails, this means that more data need to be collected, either from the same crystal (if the dose permits) or from a fresh crystal. At X06DA, the data-collection speed at 6 keV is currently limited to 2° s^−1^; data merging and substructure-determination attempts are then typically performed while collecting data. Provided that the beamline can run at very fast data-collection speeds (>10° s^−1^), a more efficient use of the beam time may consist of collecting from all crystal positions first and subsequently proceeding with merging and substructure solution.

#### 2.2.1. Strept­avidin–biotin: preparation and crystallization   

Strept­avidin from *Streptomyces avidinii* is a 52.8 kDa protein (four monomers in the asymmetric unit) with a total of 508 residues (4 × 127 residues in the asymmetric unit) and no S atoms. Strept­avidin was purchased from IBA Lifesciences. Biotin, a sulfur-containing ligand, was synthesized according to Chatterjee *et al.* (2016 ▸). The strept­avidin–biotin complex (SavB) was obtained by mixing 13.25 mg wild-type strept­avidin with 0.610 mg biotin in 500 µl H_2_O. Vapour diffusion in sitting drops was performed by mixing 1 µl SavB solution with 1 µl of a solution consisting of 80% MPD and 100 m*M* MMT pH 5.5 (Molecular Dimensions), and was equilibrated against a 500 µl reservoir containing 50% MPD. Crystals with average dimensions of 50 × 150 × 200 µm were obtained in space group *P*2_1_ within two days. The crystals were harvested and snap-cooled in liquid nitrogen for storage prior to data collection. The coordinates and structure factors of SavB from this work have been deposited in the PDB as entry 6m9b. Data-collection and refinement statistics are summarized in Table 1 ▸.

## 3. Results and discussion   

**Figure 2:**
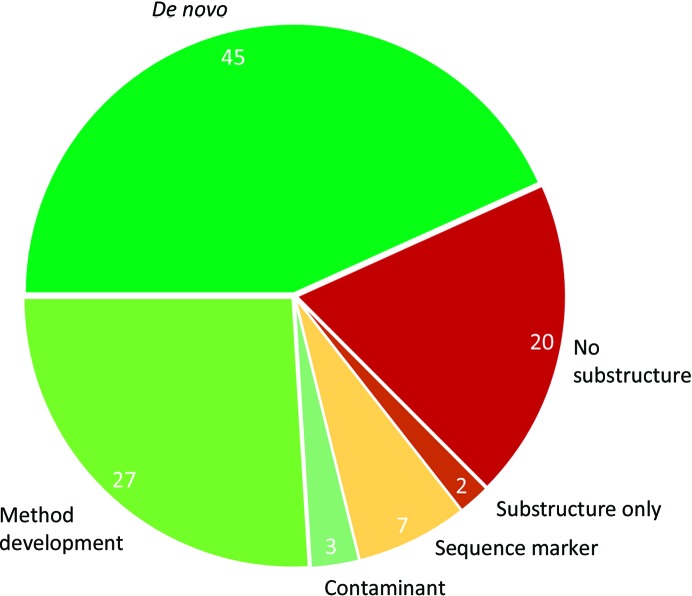
Distribution of low-energy (6 keV) experiments performed on the X06DA (PXIII) beamline in the last four years. 75 structures were successfully solved by native SAD, of which 45 were novel structures (‘*de novo*’). 27 were previously solved by other means such as molecular replacement or Se-SAD (‘method development’). In three cases, crystals of a different protein from that originally intended were obtained owing to *E. coli* contamination during purification (‘contaminant’). There were two cases where substructure solution was successful but not phasing (‘substructure only’), and 20 failed cases (‘no substructure’). Seven experiments were performed to obtain the position of the light scatterers in the structure and to help model building (‘sequence marker’).

We and our users have routinely been using our native SAD strategy for the last four years, and we have already reported some of our major successes (Weinert *et al.*, 2015 ▸; Olieric *et al.*, 2016 ▸). Based on our archive (November 2014 to January 2019), which reflects only the projects that we followed with users, we were able to determine 75 native SAD structures out of a total of 97 attempted cases. This includes the successful determination of 45 *de novo* structures (Fig. 2 ▸), of which 13 have already been published (Campagne *et al.*, 2014 ▸; Goncharenko *et al.*, 2015 ▸; Niesser *et al.*, 2016 ▸; Brandmann & Jinek, 2015 ▸; Jiang *et al.*, 2017 ▸; Ou *et al.*, 2017 ▸; Leonaitė *et al.*, 2017 ▸; Śledź & Jinek, 2016 ▸; Hermanns *et al.*, 2018 ▸; Scietti *et al.*, 2018 ▸; Hohmann *et al.*, 2018 ▸; Liu *et al.*, 2019 ▸; Wang *et al.*, 2019 ▸). Of those 97 cases, 27 structures (shown as ‘method development’ in Fig. 2 ▸) were solved to demonstrate the potential of our native SAD strategy. The majority of the 75 solved structures, provided by various users, are of high symmetry (ortho­rhombic or higher), are well diffracting (< 2.5 Å resolution) and are relatively small (<100 kDa). For such cases, a routine solution required an average of 4.5 × 360° ω scans at varying orientations and was completed in 15 min or less.

Of the 157 native SAD structures reported in the PDB, about 15% were crystallized in low-symmetry space groups (Olczak & Cianci, 2018 ▸). Meanwhile, about 30% of the total deposited structures in the PDB crystallized in monoclinic or triclinic space groups, indicating that there is a deficit of low-symmetry protein crystals that have been solved by native SAD. In fact, there are only two reports of triclinic crystal structures solved by native SAD (Banerjee *et al.*, 2016 ▸; Mueller-Dieckmann *et al.*, 2007 ▸). Out of our 97 cases, about 22% crystallized in a monoclinic space group, out of which 66% could be solved.

Because it is a *de novo* method, the data for native SAD do not rely on knowledge of the initial model, and this can lead to some surprising results. We have recently solved three such cases (Fig. 2 ▸) where users came with their desired protein crystals and could not solve the structure using molecular replacement. While early analysis of the unit-cell parameters (Berman *et al.*, 2000 ▸; McGill *et al.*, 2014 ▸; Ramraj *et al.*, 2012 ▸) or molecular-replacement searches using *Contaminer* (Hungler *et al.*, 2016 ▸) or *SIMBAD* (Simpkin *et al.*, 2018 ▸) may have been useful, they were subsequently identified as *Escherichia coli* contaminants upon collecting native SAD data at 6 keV using our strategy. They were glucosamine-6-phosphate deaminase and carbonic anhydrase in two different tetragonal crystal forms (*P*4_2_2_1_2 and *P*4_3_22).

There were two cases in which the substructure could be identified but phasing failed (Fig. 2 ▸), owing to either low resolution or low solvent content. Another 18 structures (Fig. 2 ▸) could not be determined with our native SAD protocol on account of radiation damage (crystals of <40 µm in size), low intrinsic resolution (≥4 Å), a very large substructure (>200 sites), a large size of the molecule (>300 kDa) or an unfavourable ratio between the number of observed reflections and the size of the substructure (Terwilliger *et al.*, 2016 ▸), or some combination thereof. In general, we suggest native SAD for crystals that diffract to 3 Å resolution or better. A lower resolution than this will be extremely challenging, as the anomalous signal will be even weaker.

We report on our successes with a few challenging examples below. These cases cover challenges owing to low Bijvoet ratio, low symmetry and large substructure. In addition, we describe the significance of native SAD data in identifying correct ions based on weak anomalous signal, as well as in helping in the model building of low-resolution crystal structures with sulfur anomalous peaks from both methionine and cysteine residues used as sequence markers.

### 3.1. Low Bijvoet ratio structure: strept­avidin–biotin   

**Figure 3:**
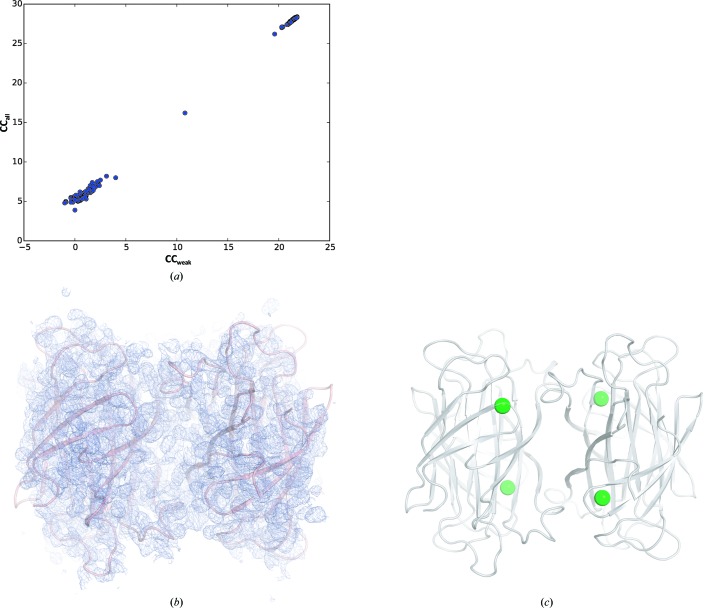
Structure determination of the strept­avidin–biotin complex by native SAD. (*a*) CC_all_ versus CC_weak_ plot, showing successful substructure determination by *SHELXD*. (*b*) Experimental phasing (2*F*_o_ − *F*_c_) map contoured at the 1.0σ level, showing the C^α^ trace after autobuilding of the model with *autoSHARP*. (*c*) Cartoon representation of the secondary structure of the SavB complex in transparent grey; four S atoms (*i.e.* anomalous scatterers) are shown as green spheres.

Native SAD can be a successful method even in particularly difficult cases where few anomalous scatterers are present. Strept­avidin, a 52.8 kDa protein (four monomers in the asymmetric unit) from *S. avidinii*, is one of these cases: this 508-residue tetrameric protein has no sulfur-containing residues nor any other significant anomalous scatterers. However, each monomer unit of strept­avidin strongly binds one molecule of biotin, a sulfur-containing ligand. In fact, the strept­avidin–biotin interaction is one of the strongest non­covalent interactions known in biology, and this turned out to be advantageous for substructure determination. The strept­avidin–biotin complex has a Bijvoet ratio of 0.6% at 6 keV, one of the lowest yet tested, and crystallizes in the monoclinic space group *P*2_1_. Using the low-dose, multi-orientation method described above, a total of 5040° (14 × 360°) of data were collected from a large (50 × 150 × 200 µm) single crystal of strept­avidin–biotin over two different areas of the crystal and at varying orientations (see Table 1 ▸ for χ/φ settings). Data processing was handled as described in Section 2.2. At 6 keV energy and a sample-to-detector distance of 120 mm, the resolution of the native SAD data collected was 2.1 Å. The large number of data proved to be critical for successful phasing but not for substructure determination; the four S atoms were easily found with about half of the total data collected (Fig. 3 ▸ *a*), but phasing proved unsuccessful without the collection of more data. Several rounds of refinement and density modification in *SHARP* (Vonrhein *et al.*, 2007 ▸) ultimately generated a density map suitable for refinement, and phase extension with *Parrot* (Cowtan, 2010 ▸) using a 360° data set collected from the same crystal at 12.4 keV improved the phase resolution to 1.55 Å (Figs. 3 ▸ *b* and 3 ▸ *c*). The phase-extended map was used for model building with *ARP*/*wARP* (Langer *et al.*, 2008 ▸), and refinement with *phenix.refine* (Afonine *et al.*, 2012 ▸) proceeded smoothly thereafter. Phase extension was not strictly necessary to finish the model building and refinement, but rather to show the utility of combining the low-energy SAD data with data collected at a higher energy to improve the resolution.

### 3.2. Large structure and low symmetry: T_2_RT-TTL and the Cas9–RNA–DNA complex   

Here, we review the protocols used for the native SAD structure determination of the largest substructures and protein complexes determined by native SAD to date: the multiprotein/multiligand tubulin T2R-TTL (Weinert *et al.*, 2015 ▸) and the Cas9–RNA– DNA complex (Olieric *et al.*, 2016 ▸). T_2_RT-TTL has a molecular mass of ∼266 kDa and contains 118 S, 13 P, three Ca^2+^ and two Cl^−^ sites in space group *P*2_1_2_1_2_1_ (Prota *et al.*, 2013 ▸). The structure was determined by applying our low-dose, multi-orientation native SAD strategy to a single crystal diffracting to around 2.3 Å resolution and with anomalous signal extending to 2.9 Å resolution. Collection and processing of each 360° ω scan was performed as described in Section 2.2. A total of eight 360° ω scans were required for successful phasing. The substructure was determined with 1000 trials of *SHELXD* at a 3.3 Å resolution cutoff and inital C^α^ traces were obtained from *SHELXE*. Model building was subsequently completed with *Buccaneer* (Cowtan, 2006 ▸).

The Cas9–RNA–DNA complex combined two challenging aspects: a large substructure and low symmetry. The molecular weight of the monomer is ∼200 kDa and the number of sites (P, S and K^+^ atoms) is 144. The protein–DNA complex crystallized in space group *C*2 (Anders *et al.*, 2014 ▸). These crystals showed an intrinsic resolution limit of ∼2.2 Å and the anomalous signal extended to 2.9 Å resolution. Adequate anomalous signal with high multiplicity at a low dose (∼0.5 MGy per data set) was achieved by combining a total of 24 data sets collected over three positions from two crystals (3 × 8 × 360° ω scans). The collection and processing of each 360° ω scan was performed as described in Section 2.2. Substructure determination was performed with 1000 trials of *SHELXD* (Sheldrick, 2010 ▸) at a 2.6 Å resolution cutoff, and initial C^α^ traces of 1060 residues (out of 1372 residues) were obtained from three cycles of auto-tracing in *SHELXE* (Sheldrick, 2010 ▸).

### 3.3. Sequence marking and ion assignment with native SAD data   

In order to visualize new structural features based on existing models, structural biologists can use low-energy data to locate light elements such as Cl^−^, K^+^ and Ca^2+^, as well as S atoms from cysteine and methionine residues, or find the phosphate backbone of DNA/RNA in the structure (Weiss *et al.*, 2001 ▸). This is particularly helpful when modelling low-resolution structures. The anomalous signals from low-*Z* scatterers are also sometimes combined with anomalous data obtained from heavy-atom derivatives or with a partial molecular-replacement model to obtain better phases (Skubák *et al.*, 2018 ▸). Recently, we have pursued seven different cases (Fig. 2 ▸) in which low-energy data were used as sequence markers, from which five structures have been published (Markovic-Mueller *et al.*, 2017 ▸; Fédry *et al.*, 2017 ▸; Engel *et al.*, 2017 ▸; Mazor *et al.*, 2017 ▸; Deneka *et al.*, 2018 ▸).

**Figure 4:**
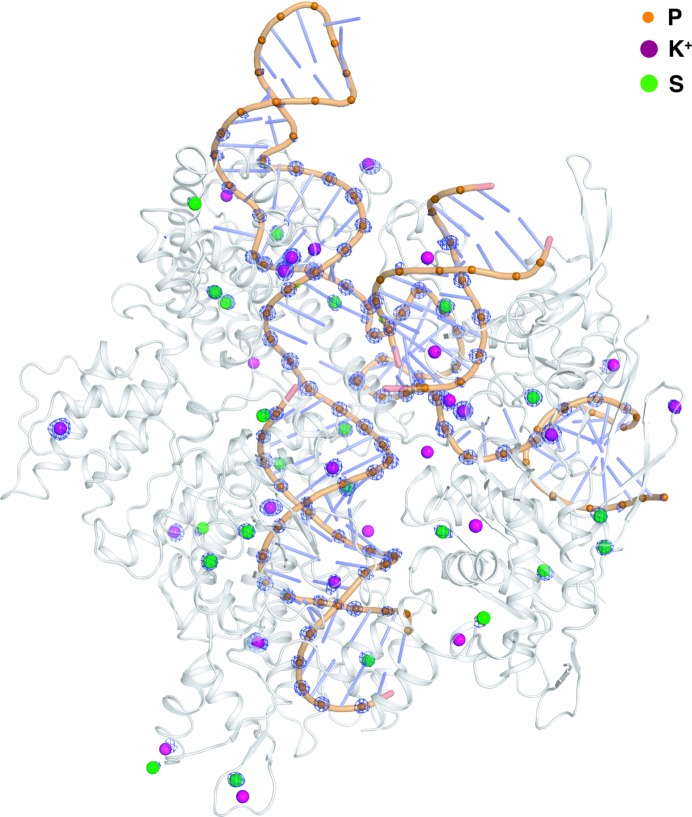
Cartoon representation of the Cas9–RNA–DNA complex with the anomalous difference Fourier map at a 4.7σ contour level. The helices from the Cas9 protein are shown in grey and the DNA backbone is shown in orange. P atoms are highlighted as orange spheres. K^+^ and S atoms are shown as magenta and green spheres, respectively. The anomalous map at the 4.7σ level is coloured in light blue, tracing the position of each scatterer.

Low-energy data can also help in the assignment of ions (Mueller-Dieckmann *et al.*, 2007 ▸; Raaf *et al.*, 2008 ▸; Liu *et al.*, 2013 ▸). There are cases in which an unknown anomalous peak from native SAD data appears at a position that was previously identified as water or some light element, providing extra information that is not available in higher-energy experiments. Sulfur and potassium are not the only possible naturally occurring anomalous scatterers: ions (for example Cl^−^, Ca^2+^, K^+^ *etc.*) that are present in the protein can also contribute to the total anomalous signal. Calcium is an especially strong anomalous scatterer at 6 keV, with *f*′′ = 2e^−^. However, while the presence of anomalous peaks at non­protein positions indicates the presence of a heavier ion, unambiguous assignment may require more information. In the case of the Cas9–RNA–DNA complex we observed anomalous peaks at the location of Mg^2+^ ions (Fig. 4 ▸), which could then be assigned as K^+^ ions using additional information from X-ray absorption spectroscopy (Olieric *et al.*, 2016 ▸). Thus, native SAD data in combination with a spectroscopic method and bond-length information, provided that a sufficiently high resolution is available, can be useful to assign correct light elemental ions, which are frequently misassigned either as water molecules or as incorrect ions.

## 4. Summary   

In the past four years, we have developed and built up a low-dose, multiple-orientation data-collection strategy for native SAD from a niche technique into a standard one that any user can easily accomplish in under 20 min. Being fast and experimentally very simple, X06DA users routinely perform experiments at 6 keV during their assigned beam time. Indeed, neither changes in the experimental setup nor prior knowledge of the crystal system are required. Advances in beamline instrumentation, in particular a highly precise multi-axis goniometer and fast photon-counting detectors, enabled these native SAD experiments. While our native SAD data-collection strategy is readily available at beamlines equipped with multi-axis goniometers, such as the popular mini-kappa (Brockhauser *et al.*, 2011 ▸), new multi-axis goniometers such as SmarGon (the multi-axis goniometer from SmarAct GmbH) are being developed and will allow full automation in multi-orientation data collection. The broad applicability, simplicity and rapidity of our strategy should encourage the structural biology community to attempt native SAD on their own as a first choice for experimental phasing: the chance of success is high, here of the order of 75–80%.

Our strategy was not sufficient in ∼20% of attempted native SAD experiments (‘no substructure’ in Fig. 2 ▸). They primarily include cases such as large substructures (>200 sites), low diffraction resolution (>3 Å) and triclinic and monoclinic space groups, as well as small crystals for which sufficient anomalous signal may not be reached within the lifetime of the crystal. In order to be successful for these cases, access to a lower X-ray energy (5–2.5 keV) may be beneficial. This demands a new type of beamline design, detectors that are better calibrated for the low X-ray energy range, and a sample environment with either a helium chamber or a vacuum. Currently, there are only two dedicated low X-ray energy beamlines in the world: BL1A at the KEK Photon Factory in Japan and the I23 beamline at the Diamond Light Source in the UK. These beamlines are equipped with advanced sample environments (*i.e.* helium or a vacuum) and special detector configurations (a V-shaped arrangement or a curved detector) to observe high-resolution reflections. This can push the limit of native SAD phasing and overcome some of the major challenges such as low diffraction resolution (Bent *et al.*, 2016 ▸). Upon considering lower X-ray energies for native SAD, X-ray absorption owing to crystal thickness is a major issue (Arndt, 1984 ▸; Wagner *et al.*, 2016 ▸; Liebschner *et al.*, 2016 ▸). This can be overcome with spherical laser-shaped crystals (Kitano *et al.*, 2004 ▸; Watanabe, 2006 ▸; Basu, Olieric *et al.*, 2019 ▸) or with micrometre-sized crystals, which are now routinely used in the field of structural biology. High-multiplicity data are challenging to obtain from micrometre-sized (<30 µm) single crystals, but it has been shown that accurate native SAD data can be gleaned from such crystals at the synchrotron using serial crystallography approaches (Huang *et al.*, 2015 ▸, 2016 ▸, 2018 ▸; Diederichs & Wang, 2017 ▸; Weinert *et al.*, 2017 ▸). Serial crystallography is a new technique, but it is maturing and expanding to experimental phasing at most modern facilities, with tunable X-ray energies, variable beam size and faster noise-free detectors (for example EIGER or JUNGFRAU), together with associated software for both data collection and processing (Axford *et al.*, 2012 ▸; Zander *et al.*, 2015 ▸; Sanishvili & Fischetti, 2017 ▸; Yamamoto *et al.*, 2017 ▸; Guo *et al.*, 2018 ▸; Basu, Kaminski *et al.*, 2019 ▸). A future promising native SAD experiment would demand a combination of the serial crystallography method with a new faster noise-free, low-energy (*i.e.* 4–2.5 keV) calibrated detector under a vacuum or helium environment.

## Supplementary Material

PDB reference: strept­avidin–biotin, 6m9b

## Figures and Tables

**Table 1:** Data-collection and processing statistics for strept­avidin–biotin (SavB) using 5.975 keV (native SAD) and 12.398 keV data obtained on the X06DA beamline at the SLS Values in parentheses are for the last shell.

	SavB, native SAD, 5.975 keV	SavB, 12.398 keV
Data collection		
Photon energy (keV)	5.975	12.398
Beam size (µm)	90 × 50	90 × 50
Flux (photons s^−1^)	1.5 × 10^10^	3 × 10^11^
Space group	*P*2_1_	*P*2_1_
*a*, *b*, *c* (Å)	50.9, 98.3, 52.7	50.9, 98.3, 52.7
α, β, γ (°)	90, 112.6, 90	90, 112.6, 90
Oscillation angle (°)	0.2	0.2
Exposure time (s)	0.1	0.1
Crystal-to-detector distance (mm)	120	120
Dose per data set (MGy)	0.54	1.64
Total oscillation (°)	Position 1, 7 × 360; position 2, 7 × 360	1 × 360
χ angles (°)	0–30; Δ = +5	0
Resolution (Å)	50–2.10 (2.19–2.10)	49.16–1.55 (1.60–1.55)
No. of unique reflections	46906 (1276)	69231 (6500)
No. of reflections	1830538 (7044)	46604 (42076)
Multiplicity	39.02 (5.52)	6.70 (6.50)
Completeness (%)	100 (100)	99.33 (93.55)
*R*_meas_ (%)	6.0 (18.30)	5.702 (61.40)
CC_1/2_ (%)	100 (97.40)	99.99 (82.10)
〈*I*/σ(*I*)〉	57.4 (7.38)	22.30 (2.95)
Mosaicity (°)	0.18	0.18
No. of anomalous scatterers	4	N/A
Resolution cutoff for *SHELXD* (Å)	2.6	N/A
Correlation coefficient (all/weak)	28.32/21.66	N/A
Solvent content (%)	46.6	49.8
Refinement		
Resolution (Å)		49.16–1.55 (1.60–1.55)
*R*_work_ (%)		15.36 (23.70)
*R*_free_ (%)		18.34 (26.34)
No. of non-H atoms		
Total		4187
Macromolecules		3617
Ligands		64
Water		506
No. of protein residues		477
R.m.s. deviations		
Bond lengths (Å)		0.009
Bond angles (°)		1.37
Wilson *B* (Å^2^)		16.73
Average *B* (Å^2^)		
Overall		24.90
Macromolecules		23.40
Ligands		15.00
Water		36.70
Clashscore		1.67
Ramachandran plot		
Favoured (%)		98
Allowed (%)		2
Outliers (%)		0
PDB code		6m9b
